# Antibodies targeting the neuraminidase active site inhibit influenza H3N2 viruses with an S245N glycosylation site

**DOI:** 10.1038/s41467-022-35586-7

**Published:** 2022-12-21

**Authors:** Daniel Stadlbauer, Meagan McMahon, Hannah L. Turner, Xueyong Zhu, Hongquan Wan, Juan Manuel Carreño, George O’Dell, Shirin Strohmeier, Zain Khalil, Marta Luksza, Harm van Bakel, Viviana Simon, Ali H. Ellebedy, Ian A. Wilson, Andrew B. Ward, Florian Krammer

**Affiliations:** 1 Department of Microbiology, Icahn School of Medicine at Mount Sinai, New York, NY USA; 2 Department of Integrative Structural and Computational Biology, The Scripps Research Institute, La Jolla, CA USA; 3 Division of Viral Products, Center for Biologics Evaluation and Research, Food and Drug Administration, Silver Spring, MD USA; 4 Department of Biotechnology, University of Natural Resources and Life Sciences, Vienna, Austria; 5 Department of Genetics and Genomic Sciences, Icahn School of Medicine at Mount Sinai, New York, NY USA; 6 Department of Oncological Sciences, Icahn School of Medicine at Mount Sinai, New York, NY USA; 7 Icahn Genomics Institute, Icahn School of Medicine at Mount Sinai, New York, NY USA; 8 Global Health Emerging Pathogens Institute, Icahn School of Medicine at Mount Sinai, New York, NY USA; 9 Department of Pathology, Molecular and Cell Based Medicine, Icahn School of Medicine at Mount Sinai, New York, NY USA; 10 Center for Vaccine Research and Pandemic Preparedness (C-VaRPP), Icahn School of Medicine at Mount Sinai, New York, NY USA; 11 Division of Infectious Diseases, Department of Medicine, Icahn School of Medicine at Mount Sinai, New York, NY USA; 12 Division of Immunobiology, Department of Pathology and Immunology, Washington University School of Medicine, St. Louis, MO USA; 13 The Skaggs Institute for Chemical Biology, The Scripps Research Institute, La Jolla, CA USA

**Keywords:** Influenza virus, Antibodies

## Abstract

Contemporary influenza A H3N2 viruses circulating since 2016 have acquired a glycosylation site in the neuraminidase in close proximity to the enzymatic active site. Here, we investigate if this S245N glycosylation site, as a result of antigenic evolution, can impact binding and function of human monoclonal antibodies that target the conserved active site. While we find that a reduction in the inhibitory ability of neuraminidase active site binders is measurable, this class of broadly reactive monoclonal antibodies maintains protective efficacy in vivo.

## Abstract (web summary)

Antibodies that broadly inhibit influenza virus neuraminidase by binding to its active site could be therapeutic candidates, but circulating viruses have acquired a glycosylation site in that region. Here, the authors show that, while the S245N glycosylation site affects binding of tested monoclonal antibodies, protective activity in a mouse model is maintained.

## Introduction

H1N1, H3N2, and type B seasonal influenza viruses cause a large number of infections and deaths annually^1^. Typically, influenza seasons that are dominated by H3N2 viruses are worse in terms of disease outcome, number of hospitalizations, and are especially concerning for at-risk populations such as the elderly or younger children^2^. In recent years, matching the H3N2 vaccine component to actual circulating H3N2 viruses has been particularly challenging due to the occurrence of mutations in the hemagglutinin (HA) during the vaccine production process in eggs^3,4^ and the emergence of genetically different co-circulating H3N2 clades^5^. In addition, the introduction of an *N*-linked glycosylation site at residue 245 of the neuraminidase (NA) contributed to antigenic drift^6^ and resulted in the blocking of monoclonal and human serum NA-specific antibodies^7^. Therefore, broadly protective, universal influenza vaccines^8^ or improved classical seasonal vaccine formulations are urgently needed^9^. Such efforts are informed by the structural and functional characterization of antibodies that target broadly neutralizing epitopes on the influenza virus NA. A double mutation in the NA of influenza A H3N2 viruses, which leads to changes in amino acids at positions 245 and 247, was first detected in 2014 and, by 2016/17, almost all circulating H3N2 viruses in humans carried these mutations that introduced an *N*-glycosylation site at Asn245, as mentioned above^6^. These mutations have become fixed in circulating H3N2 viruses and the introduced glycan might then contribute to the shielding of certain epitopes.

**Fig. 1:**
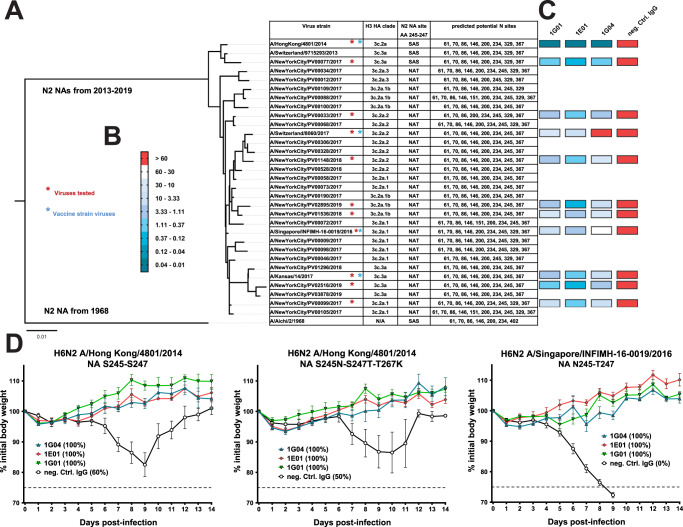
Phylogenetic tree of N2 NA of H3N2 viruses and functional characterization of NA-specific antibodies. **A** Phylogenetic tree of N2 NAs. The scale bar represents a 1% change in nucleotides. The tree was built using nucleotide sequences in ClustalOmega and visualized in FigTree. Vaccine virus strains and other strains tested in this study are indicated. The H3N2 clade (based on HA), the amino acid residues at position 245–247, and all predicted potential glycosylation sites are shown. **B** Scale bar for the IC_50_ in μg/ml of viruses tested as shown in the heatmap. **C** Heatmap of IC_50_ of three NA antibodies and irrelevant IgG control mAb. IC_50_ values shown were measured in ELLAs except for viruses A/New York City/PV00033/2017 and A/New York City/PV00077/2017, were the inhibitory concentrations were determined in an NA-Star assay because of low NA activity in the ELLA. The antibodies were tested against the viruses in duplicate and the average of the two measurements was used to calculate the IC_50_ values depicted. **D** Weight loss curves of DBA/2 J mice challenged with reassortant H6N2 viruses. The data are presented as the mean of the group (*n* = 5 per group, except for the middle panel where the *n* in the irrelevant control IgG group was 4) with error bars indicating the standard error of the mean. Percent survival upon virus challenge is indicated in parenthesis and the dashed line represents 75% of the initial body weight, which was defined as the humane endpoint. The experiment was performed once. Source data are provided as a Source Data file.

Here, we test three previously described broadly protective influenza virus NA monoclonal antibodies (mAbs)^10^ against a panel of H3N2 viruses, including influenza virus vaccine strains and clinical H3N2 isolates (Fig. 1A and Supplementary Fig. 1). We show, that a reduction in the inhibitory ability of these mAb active site binders is measurable against viruses harboring the Asn245 glycosylation site but that this class of broadly reactive antibodies maintains protective efficacy in vivo.

## Results

### Enzymatic-site targeting mAbs recognize and inhibit drifted N2 NA

We found that the three NA mAbs could bind to recombinant N2 NAs (produced in the baculovirus system) of three recent influenza virus vaccine strains that harbor the S245N glycosylation site in enzyme-linked immunosorbent assays (ELISAs) (Supplementary Fig. 2) and that the antibodies can inhibit the NA enzymatic activity of vaccine strain viruses (grown in eggs) as measured in enzyme-linked lectin assays (ELLAs) (Fig. 1C, Supplementary Fig. 2, and Source Data file). Further, the three antibodies inhibited, to different extents, the NA activity of various clinical H3N2 virus isolates (grown in cell culture) that were cultured from nasopharyngeal swab specimen obtained from patients seeking care at the Mount Sinai Hospital in the years 2017 to 2019 (Fig. 1C). Here we selected antigenically distinct clinical virus isolates based on the N2 neuraminidase sequence for testing to cover a broad range of circulating H3N2 viruses. The 50% inhibitory concentration (IC_50_) values of the antibodies tested against viruses without the glycan [A/Hong Kong/4801/2014 (Hong Kong14) and  A/New York City/PV00077/2017] were lower (0.01-0.69 ug/mL)) compared to viruses that harbor the glycan (0.60–35.62 ug/mL). Antibody 1E01 had the lowest IC_50_ across the viruses tested, followed by 1G01, which was previously shown to be the most cross-reactive of the three antibodies, and 1G04 (Fig. 1C). Interestingly, in some cases, differences between mAbs become apparent only in the NI assay but not in the ELISA. This could be due to differences in glycan size between recombinant proteins produced in insect cells and viruses grown in cells or embryonated eggs^11^.

### 1G01-like mAbs maintain in vivo protection against drifted N2 NA

Next, we investigated the protective efficacy of the antibodies in passive transfer and viral challenge experiments in DBA/2 J mice using reassortant viruses that all contain the same avian H6 HA but different N2 NAs. The first virus carried the wild-type N2 NA of the Hong Kong14 virus (no glycan at position 245). The second virus expressed the Hong Kong14 N2 NA but with amino acid changes at positions 245 (S245N) and 247 (S247T) that introduce a glycosylation site and a change at some distance from the catalytic active site at position 267 (T267K) that is also present in the N2 NA of A/Singapore/INFIMH-16-0019/2016 (Singapore16). The third virus was an H6 reassortant virus with the N2 NA of Singapore16 that harbors the glycosylation site naturally^6^. To confirm the presence of the additional glycan on the Singapore16 N2 NA expressing virus grown in eggs or cell culture, peptide:*N*-glycosidase F (PNGaseF) treatment followed by sodium dodecyl sulfate–polyacrylamide gel electrophoresis (SDS-PAGE) and Western blotting was performed (Supplementary Fig. 3). The SDS-PAGE indicates the presence of an additional glycosylation site on the Singapore16 N2 NA when compared to the Hong Kong14 N2 NA, and PNGaseG treatment removes glycans allowing the NA proteins to run at the same size on the SDS-PAGE. These differences in glycosylation sites are similar to those previously shown by others^6,12^.

The three antibodies were given intraperitoneally at a concentration of 5 mg/kg 2 h prior to challenge and conferred full protection against morbidity (Hong Kong14 virus without or with artificially introduced 245 glycosylation site) or mortality (Singapore16 virus with glycosylation site) (Fig. 1D) compared to the control groups. Interestingly, the functional capacity of the antibodies in vitro in ELLAs displayed stark differences between the viruses with or without the glycan (Supplementary Fig. 4) that were not recapitulated in the mouse model. This finding could be explained by the contribution of Fc-effector functions, such as antibody-dependent cellular cytotoxicity (ADCC) or antibody-dependent cellular phagocytosis (ADCP), to protective efficacy in vivo, by differences in how the epitope is displayed in the respective test system, or could indicate that only very low neuraminidase inhibition (NI) activity is needed for protection in vivo. Furthermore, the glycan at NA Asn245 has been shown to reduce NA enzymatic activity^7^. The reduced activity is controlled for in the in vitro experiments to measure IC_50_s; however, reduced NA activity in the in vivo experiments might be another reason for the discordance of measured IC_50_s and in vivo protective effects.

### Structural characterization of the mAb-drifted N2 interaction

**Fig. 2:**
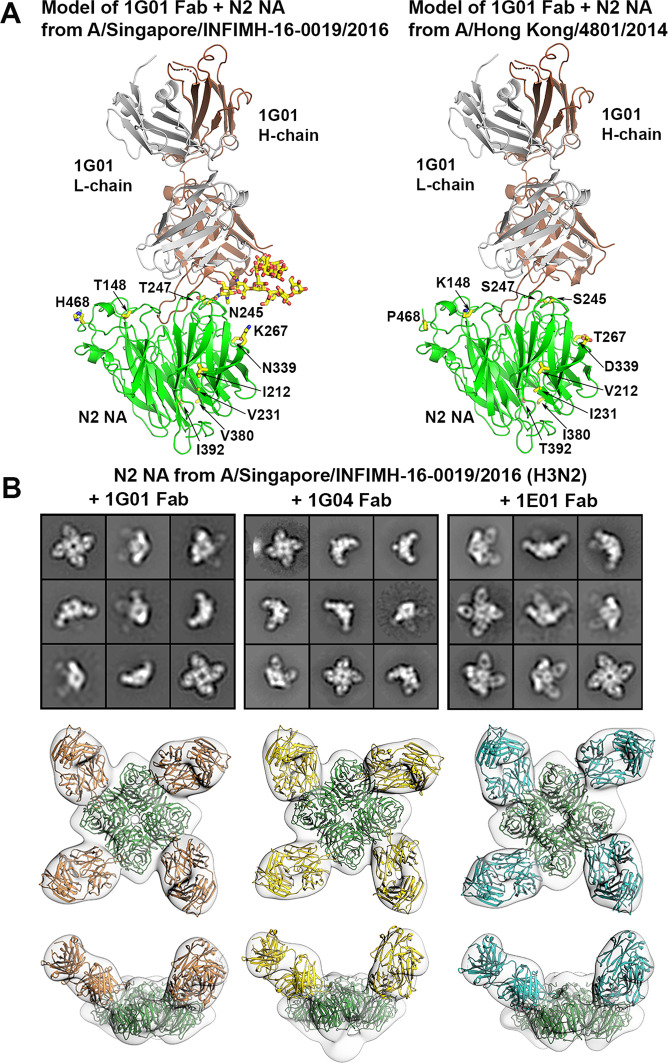
Model of the complex of Singapore16 and Hong Kong14 N2 NAs with 1G01 Fab and negative-stain electron microscopy of antibodies 1G01, 1G04, and 1E01 bound to Singapore16 N2 NA. **A** The Singapore16 N2 NA was modeled with a mannose-9 glycan at Asn245. The 1G01 Fab is able to interact with the N2 NA (left). The glycan can be accommodated without steric clash with the 1G01 Fab. A model of the interaction of 1G01 Fab to Hong Kong14 N2 NA is also shown (right). NA protomers are colored green. Asn245 and attached glycans, as well as the ten residues that are not conserved between these two N2 NAs in their ectodomain (82–469) are shown in sticks and colored with yellow carbon, red oxygen and blue nitrogen atoms. The 1G01 Fab is colored brown (heavy chain) and gray (light chain). **B** Two-dimensional class averages of Singapore16 N2 NA in complex with 1G01, 1G04, and 1E01 Fabs (Top). Standard view (middle) and side view (bottom) of three-dimensional reconstruction of negative-stain EM maps of Singapore16 N2 NA tetramer (center, in green) in complex with four 1G01 Fabs (in orange), four 1G04 Fabs (in yellow), and 1E01 Fabs (in cyan), respectively (densities with a dimple on the periphery). The crystal structures of 1G01 Fab (PDB code 6Q23), 1G04 Fab (PDB code 6Q1Z), and 1E01 Fab (PDB code 6Q20) and the Singapore16 N2 NA tetramer model were docked into the reconstruction, respectively, and the Fabs were adjusted individually to best fit the EM density.

To illustrate the location of the glycan in the context of the antigen–antibody interaction, we modeled the complex of the Singapore16 N2 NA with the antibody binding fragment (Fab) of 1G01. Based on the modeling results in combination with the binding, NI and protection data, we concluded that 1G01 is still able to bind to Singapore16 N2 NA with the additional Asn245 glycosylation (Fig. 2A), although the glycan attached at NA Asn245 could potentially form transient interactions with 1G01. As a control, we also modeled the complex of the Hong Kong14 N2 NA with 1G01 Fab (Fig. 2A), where the only difference in the 1G01 epitope was at N245 and T247 that encodes the 245 glycosylation site in Singapore16 N2 NA as compared to S245 and S247 in Hong Kong14 N2 NA. Similarly, antibodies 1E01 and 1G04 also appear able to bind Singapore16 N2 NA with the additional Asn245 glycosylation (Supplementary Fig. 5). Further, we confirmed the modeling results by performing negative-stain electron microscopy (nsEM) studies (Fig. 2B and Supplementary Fig. 6). Examination of 2D class averages from the nsEM images for complexes of Singapore16 N2 NA with Fabs 1G01, 1G04, or 1E01 showed that one Fab bound to the active site of each N2 NA protomer (i.e., four Fabs per NA tetramer) (Fig. 2B). In 3D reconstructions, antibodies 1G01, 1G04, and 1E01 were found to bind to Singapore16 N2 NA in a similar manner to binding to N1 NA from A/California/04/2009 (H1N1) (Cali04), Hunan16 N9 NA from A/Hunan/02650/2016 (H7N9) (Hunan16), and N2 NA from A/Japan/305/1957 (H2N2) (Japan57)^10^, respectively, in which the 1G01, 1G04, and 1E01 epitopes consisted mostly of conserved active site residues of the NAs (Fig. 2B). Sequence comparison of the 1E01 epitope in Japan57 N2 NA, as well as the 1G01 epitope in Cali04 N1 NA and the 1G04 epitope in Hunan16 N9 NA, with Hong Kong14 N2 NA and Singapore16 N2 NA indicated that the their epitopes are quite conserved in these N2 NAs (Supplementary Figs. 7, 8, 9). Notwithstanding, the epitope differences between these three antibodies to N2 NA (Supplementary Figs. 7, 8, 9), the potential different angles of the approach of these antibodies to N2 NA (Supplementary Fig. 10), as well as the sequence differences between these antibodies^10^, may contribute to the differences in their binding to NA and NA inhibition efficacy (Fig. 1 and Supplementary Figs. 1, 2, 4).

## Discussion

In summary, we demonstrate that broadly protective human antibodies that target the active site of the influenza virus NA can maintain binding, functionality, and protective efficacy against contemporary H3N2 viruses with an S245N glycosylation site. In previous studies, antigenic drift was observed in the NAs of recent viruses with a reduction in NI titers of human sera against these NAs and a loss of in vivo protection in passive transfer studies; substantially reduced binding of NA-specific mAbs was also demonstrated^6^. However, the introduction of the S245N glycan into the N2 NA did not prevent the binding of 1G01 and 1G01-like mAbs that directly target the NA active site, as confirmed by electron microscopy. Of note, NI activity of human polyclonal serum was also not completely abrogated, although significantly reduced, in earlier studies, perhaps suggesting the presence of 1G01-like antibodies in serum that maintain activity. Of course, the inhibiting antibody response to NA is not restricted to the active site and antibodies to other epitopes may contribute to this residual activity. Our findings showing the continued activity of 1G01-like antibodies against drifted N2 NA, in combination with the exclusive breadth across NA subtypes, further highlight the value of these particular antibodies as possible prophylactic or therapeutic agents. In addition, developing vaccines that can elicit 1G01-like antibodies would allow for protection against drifted viruses and broad protection in general.

## Methods

Our research conforms to all ethical regulations. Specifically, animal experiments were conducted in compliance with protocols approved by the Icahn School of Medicine at Mount Sinai’s Institutional Animal Care and Use Committee.

### Cells, proteins, and viruses

Madin Darby canine kidney (MDCK, source ATCC) cells were grown in Dulbecco’s modified Eagle’s medium (DMEM; Gibco) containing FBS (fetal bovine serum, 10%; Corning) and penicillin–streptomycin antibiotics mix (pen-strep antibiotics mix, 100 μg/mL streptomycin, 100 U/mL of penicillin; Gibco) resulting in complete DMEM (cDMEM). Human embryonic kidney (HEK) cells (293 T, source ATCC) were grown and maintained in cDMEM. HEK 293 F cells (Thermo Fisher) were grown in Expi293 Expression medium (Gibco). BTI-*TN*−5B1- 4 cells (*Trichoplusia ni*, source Vienna Institute of Biotechnology, University of Natural Resources and Life Sciences) were grown in serum-free SFX medium (HyClone) supplemented with pen-strep antibiotics mix . Sf9 cells (*Spodoptera frugiperda*, source Vienna Institute of Biotechnology, University of Natural Resources and Life Sciences) were maintained in TNM-FH medium (Gemini Bio-Products) in the presence of pen-strep antibiotics mix and 10% FBS. Vaccine strains and reassortant viruses were grown in 8–10-day-old embryonated chicken eggs (Charles River Laboratories) at 37 °C (influenza A viruses) for 2 days. Virus reassortants used in this study were rescued by plasmid-based reverse genetic techniques as previously described in refs. 6, 13. All reassortant viruses bear the internal genes from A/Puerto Rico/8/1934 H1N1. Primary virus isolates were obtained from discarded nasopharyngeal specimens that tested positive for influenza virus infection. Briefly, specimens were pre-diluted in infection media consisting of Minimum Essential Media (Gibco) supplemented with 1 µg/ml of tosylsulfonyl phenylalanyl chloromethyl ketone (TPCK)-treated trypsin (Gibco) and added to 90% confluent MDCK cell-monolayers. Infections were let to proceed for 1 h with intermittent shaking. The inoculum was removed and infection media was added. Plates were incubated at 37 °C with 5% CO_2_ and supernatants were collected at 24–48 hpi when a cytopathic effect was detected. Samples were cleared by centrifugation at 4300 × *g* for 5 min and aliquoted through 0.22 µm pore size membrane filters (Millipore). Supernatants were stored at −80 °C until further use and viruses were sequenced after culture. To purify viruses for binding assays, viruses were grown in eggs or in cell culture and purified over a 30% sucrose cushion.

Recombinant proteins were expressed in the baculovirus expression system as previously described in detail in ref. 14. All NAs were expressed as ectodomains with an *N*-terminal vasodilator-stimulated phosphoprotein (VASP) tetramerization domain and a hexahistidine tag for purification.

### Monoclonal antibody generation and purification

Heavy and light chain plasmids were co-transfected into Expi293F cells for expression and antibodies were purified via sepharose G columns (GE Healthcare)^10^. The antibody sequences have been uploaded on GenBank (accession numbers: MN013068 (1G01 VH), MN013072 (1G01 Vκ), MN013070 (1E01 VH) MN013070 (1E01 Vκ), MN013069 (1G04 VH), and MN013073 (1G04 Vκ). The heavy and light chain plasmids of an irrelevant human IgG control monoclonal antibody were co-transfected and purified similarly.

### Enzyme-linked immunosorbent assay

Microtiter 96-well plates (Thermo Fisher) were coated with 50 μL recombinant NA at a concentration of 2 μg/mL in phosphate-buffered saline (PBS; Gibco) at 4 °C overnight. The following day, 220 μL blocking solution (PBS (Gibco) supplemented with 0.1% Tween 20 (Fisher Scientific), 3% goat serum (Life Technologies), and 0.5% milk powder (American-Bio)) was added to all wells and the plates were incubated for 1 h at room temperature. The antibodies were diluted to a starting concentration of 30 μg/mL, serially diluted 1:3 in blocking solution, and incubated for 2 h in a room temperature incubator. The microtiter plates were washed three times with T-PBS (PBS (Gibco) supplemented with 0.1% Tween 20 (Fisher Scientific)) and 50 μL goat anti-human IgG (Fab specific) horseradish peroxidase antibody (HRP; Sigma, #A0293) diluted 1:3000 in blocking solution was added to all wells and incubated for 1 h at room temperature. The plates were washed four times with shaking and 100 μL SigmaFast o-phenylenediamine dihydrochloride (OPD; Sigma) was added to all wells. After 10 min, the reaction was stopped with 50 μL 3 M hydrochloric acid (Thermo Fisher) and the plates were read at a wavelength of 490 nm with a plate reader (BioTek). The data were analyzed in Microsoft Excel and GraphPad Prism 7. The data were visualized as binding curves by applying a nonlinear fit.

### Enzyme-linked lectin assay

Ninety-six-well microtiter plates (Thermo Fisher) were coated with 100 μL/well fetuin (Sigma) at a concentration of 25 μg/mL in PBS at 4 °C overnight. To determine the amount of virus to use in the assay, the viruses were serially diluted twofold in sample diluent (PBS (Gibco) with 0.9 mM CaCl_2_ and 0.5 mM MgCl_2_ supplemented with 1% bovine serum albumin (MP Biomedicals) and 0.5% Tween 20 (Fisher Scientific)) in cell culture 96-well plates. The fetuin plates were washed three times with T-PBS and 100 ul of the diluted viruses transferred into the fetuin plates. The plates were incubated for 18 h at 37 °C and washed three times with T-PBS and HRP-conjugated peanut agglutinin (PNA) in PBS was added (100 ul/well) to the plates. The plates were incubated at RT for 2 h and washed four times with shaking and developed with SigmaFast OPD. Absorbance was read at 490 nm on a microplate reader. The data were analyzed using GraphPad Prism 8 and the 50% effective concentration (EC_50_) was calculated based on four-parameter nonlinear regression. The dilution of the virus that resulted in 2x the EC_50_ was chosen for the subsequent NA inhibition assay.

To measure the inhibitory concentration of the antibodies, the antibodies were serially diluted in sample diluent (starting concentration 30 ug/ml) and incubated for 18 h at 37 °C with an equal volume (50 ul) of the selected virus dilution in the fetuin-coated plates. The remainder of the assay was performed as described above. One column on the plate contained sample diluent without antibodies and served as a positive (virus-only) control. Another column contained sample diluent only (no virus) and served as negative (background) control. Data were analyzed in GraphPad Prism 8 and the 50% inhibitory concentration (IC_50_) was calculated based on nonlinear regression using a four-parameter curve fit.

### NA-Star assay

The NA-Star assay was performed for viruses that had low NA activity in the ELLA. The NA-Star can measure NA activity with higher sensitivity. The antibodies tested in the present study target the NA active site directly, which makes it possible to use the NA-Star assay for the determination of NAI. Typically, the NA-Star kit is used to detect resistance to neuraminidase inhibitors (e.g. small molecules). The NA-Star Influenza Neuraminidase Inhibitor Resistance Detection Kit (Applied Biosystems) was used according to the manufacturer’s protocol. In brief, the antibodies were diluted to a concentration of 30 ug/ml, serially diluted twofold in assay buffer, and 25 ul transferred to a white 96-well cell culture plate. Twenty-five ul of virus diluted to 2xEC_50_ (as determined in prior assays testing the respective viruses in the absence of antibodies) was added to each well and the plates were incubated for 20 min at 37 °C with shaking. The NA-Star substrate was prepared immediately before use and 10 ul/well was added to all wells. The plates were incubated for 30 min at RT and 60 ul/well of NA-Star accelerator solution was added shortly before reading the plates using a microtiter plate reader (BioTek). The data were analyzed in GraphPad Prism 8 and the inhibition curves were plotted.

### PNGaseF treatment, SDS-PAGE, and Western blot

Purified viruses were treated using the PNGaseF kit (NEB) according to the manufacturer’s instructions. The samples were then treated with 4X-Laemli buffer (Bio-Rad) containing beta-mercaptoethanol (BME) and heated at 100 °C for 5 min. Approximately 1 ug purified virus was added to each lane on a 7.5% Mini-PROTEAN® TGX™ precast protein gel (Bio-Rad) and run for 5.5 h at 25 V. After electrophoresis, the proteins were transferred to a nitrocellulose membrane using the iBlot transfer and stack device (Invitrogen) at 20 V for 7 min. The membranes were blocked in 5% nonfat dry milk (Bio-Rad) in PBS-T (0.5%) for 1 h at room temperature with shaking. The blocking solution was removed and anti-N2 guinea pig antisera (generated in-house by vaccinating guinea pigs intramuscularly with Hong Kong14 N2 recombinant protein) diluted 1:2000 in PBS supplemented with 1% (w/v) BSA was added. The membranes were incubated overnight and washed three times with PBS-T. The following day, a donkey anti-guinea pig IgG-horseradish peroxidase conjugate (IgG-HRP; EMD Millipore, AP193P) was added for 1 h at room temperature and the membranes were washed. The membranes were developed by adding ECL prime and incubated for 5 min. The developed blot membranes were visualized by scanning using an ImageScanner III imager with accompanying LabScan software (GE Healthcare).

### Passive transfer experiments in mice

Animal experiments were conducted in compliance with protocols approved by the Icahn School of Medicine at Mount Sinai’s Institutional Animal Care and Use Committee. Mice were housed in a facility with a 12 h light/dark cycle, room temperature, relative humidity between 20 and 30% and access to food ad libitum. Passive transfer experiments to test the prophylactic efficacy of the mAbs were performed as described earlier^10^. In brief, 6*–*8 weeks-old female DBA/2 J mice (*n* = 5 mice/group except for Fig. 1D in the middle panel in the irrelevant IgG control where *n* = 4; mice were sourced from The Jackson Laboratory) were given antibody 1G01, 1E01, and 1G04 at a concentration of 5 mg/kg or an irrelevant human IgG control antibody at the same dose intraperitoneally in a volume of 100 ul. Two hours post transfer, the mice were anesthetized with a ketamine-xylazine-water mixture (0.15 mg ketamine/kg and 0.03 mg/kg xylazine of body weight; 100 μl intraperitoneally) and challenged intranasally with 10 × the 50% mouse lethal dose (mLD_50_) of the respective challenge virus. H6N2 reassortant viruses were used in this study because of their ability to cause disease in the DBA/2 J mouse model. Weight loss was monitored daily for 14 days and the humane endpoint was defined as a loss of 25% of the initial day 0 body weight. The data were recorded in Microsoft Excel and the weight loss and survival graphs were visualized in GraphPad Prism 8.

### Modeling of Singapore16 and Hong Kong14 N2 NAs in complex with 1G01 Fab

The head domains of N2 NAs (residues 82–469) from A/Singapore/INFIMH-16-0019/2016 (H3N2) (Singapore16 N2, GISAID^14^ accession number EPI810155) and A/Hong Kong/4801/2014 (H3N2) (Hong Kong14 N2, GISAID accession number EPI675797) were modeled by MODELER^15^ using a crystal structure of the N2 NA from A/Tanzania/205/2010 (H3N2) (PDB ID 4GZO, 2.6 Å resolution^16^) as a template, with which Singapore16 N2 NA and Hong Kong14 N2 NA share 97.4 and 98.5% amino acid identity, respectively. A model of the complex between Singapore N2 NA and 1G01 Fab was constructed in Coot^17^ using the crystal structure of the N1 NA from A/California/04/2009 (H1N1) (Cali04 N1) in complex with 1G01 (PDB ID 6Q23, 3.27 Å resolution^10^) as a template by superimposing the model of Singapore N2 NA to the Cali04 N1 NA; a mannose-9 glycan was modeled at Asn245 for illustration, although the glycosylation type (high mannose or complex) is not known. A model of the complex between Hong Kong14 N2 NA and 1G01 Fab was also constructed in Coot^17^ as above by superimposing the model of Hong Kong14 N2 NA to the Cali04 N1 NA. Furthermore, models of the complexes between Singapore16 N2 NA and 1E01 or 1G04 Fabs were also constructed in Coot^17^ as above using the crystal structures of 1E01 Fab with N2 NA from A/Japan/305/1957 (H2N2) (Japan57, PDB ID 6Q20, 2.45 Å resolution^10^) and 1G04 Fab with N9 NA from A/Hunan/02650/2016 (H7N9) (Hunan16, PDB ID 6Q1Z, 3.45 Å resolution^10^), respectively.

### Negative stain electron microscopy studies of Singapore16 N2 in complex with broadly-protective anti-NA antibodies 1G01, 1G04, and 1E01

The head domain of the N2 NA (residues 82–469) from Singapore16 with a VASP tetramerization domain was cloned and expressed using the baculovirus system in Sf9 cells essentially as previously described in ref. 18. Singapore16 N2 NA without VASP tetramerization domain was obtained by thrombin cleavage of the purified Singapore16 N2 NA with the VASP tetramerization domain. Broadly protective anti-NA antibody Fabs 1G01, 1G04, and 1E10 were expressed in mammalian ExpiCHO cells as previously reported^10^.

Antibody 1G01 and 1E01 Fabs were added in 4 M excess to Singapore16 N2 NA without the VASP tetramerization domain, and 1G04 Fab was added in 4 molar excess to Singapore16 N2 NA with the VASP tetramerization domain. The N2-Fab complexes were incubated overnight at 4 °C and directly added to 400 mesh carbon-covered copper grids and stained with 2% uranyl formate. The grids were imaged on a Tecnai TF20 equipped with a TVIPS 4k × 4k camera or a Talos Arctica single tilt equipped with a 4k × 4k Ceta camera. Micrographs were collected using Leginon^19^ and uploaded to Appion^20^ for processing. Particles were picked using DoGpicker^21^ and stacked particles were put into Relion^22^ for 2D classification and 3D classification. Chimera^23^ was used to dock the individual crystal structures of Fabs 1G01 (PDB code 6Q23), 1G04 (PDB code 6Q1Z), and 1E10 (PDB code 6Q20) and the Singapore16 N2 NA tetramer model into 3D EM maps.

### NA sequences

The sequences for the generation of the phylogenetic tree were downloaded from the Influenza Resource Database (www.fludb.org) or the Global Initiative on Sharing Avian Influenza Data (www.gisaid.org). The primary H3N2 virus isolates were sequenced in-house and the sequences were provided by the Mount Sinai Pathogen Surveillance Program. These were then uploaded to GISAID and Genbank (see below).

### Reporting summary

Further information on research design is available in the Nature Portfolio Reporting Summary linked to this article.

## Supplementary information

Supplementary Information

Reporting Summary

## Data Availability

Data supporting our work are available in the paper and Supplementary Figs. 1–10. Source data are provided with this paper. Virus sequences used are available the Global Initiative on Sharing Avian Influenza Data (www.gisaid.org and/or at Genbank): A/New York City/PV00077/2017 (EPI_ISL_4073021, NA:MW651812,NP:MW651813,NS:MW651814,PB2:MW651815,HA:MW651816,PA:MW651817,M:MW651818,PB1:MW651819), A/New York City/PV00012/2017 (EPI_ISL_4062122, PB2:OP872033,PB1:OP872034,PA:OP872035,HA:OP872036,NP:OP872037,NA:OP872038,M:OP872039,NS:OP872040), A/New York City/PV00034/2017 (EPI_ISL_15842142, PB2:OP871889,PB1:OP871890,PA:OP871891,HA:OP871892,NP:OP871893,NA:OP871894,M:OP871895,NS:OP871896), A/New York City/PV00109/2017 (EPI_ISL_15842162, PB2:OP871905,PB1:OP871906,PA:OP871907,HA:OP871908,NP:OP871909,NA:OP871910,M:OP871911,NS:OP871912), A/New York City/PV00088/2017 (EPI_ISL_15842180, PB2:OP871913,PB1:OP871914,PA:OP871915,HA:OP871916,NP:OP871917,NA:OP871918,M:OP871919,NS:OP871920), A/New York City/PV00100/2017 (EPI_ISL_15842199, PB2:OP871977,PB1:OP871978,PA:OP871979,HA:OP871980,NP:OP871981,NA:OP871982,M:OP871983,NS:OP871984), A/New York City/PV00033/2017 (EPI_ISL_4073067, NA:MW651820,M:MW651821,PB2:MW651822,NS:MW651823,PA:MW651824,PB1:MW651825,NP:MW651826,HA:MW651827), A/New York City/PV00068/2017 (EPI_ISL_15842216, PB2:OP871921,PB1:OP871922,PA:OP871923,HA:OP871924,NP:OP871925,NA:OP871926,M:OP871927,NS:OP871928), A/New York City/PV00306/2017 (EPI_ISL_15842235, PB2:OP872025,PB1:OP872026,PA:OP872027,HA:OP872028,NP:OP872029,NA:OP872030,M:OP872031,NS:OP872032), A/New York City/PV00328/2017 (EPI_ISL_15842254, PB2:OP871985,PB1:OP871986,PA:OP871987,HA:OP871988,NP:OP871989,NA:OP871990,M:OP871991,NS:OP871992), A/New York City/PV01148/2018 (EPI_ISL_326910, PB2:OP872017,PB1:OP872018,PA:OP872019,HA:OP872020,NP:OP872021,NA:OP872022,M:OP872023,NS:OP872024), A/New York City/PV00528/2018 (EPI_ISL_326929, PB2:OP871969,PB1:OP871970,PA:OP871971,HA:OP871972,NP:OP871973,NA:OP871974,M:OP871975,NS:OP871976), A/New York City/PV00058/2017 (EPI_ISL_15842270, PB2:OP871937,PB1:OP871938,PA:OP871939,HA:OP871940,NP:OP871941,NA:OP871942,M:OP871943,NS:OP871944), A/New York City/PV00073/2017 (EPI_ISL_15842280, PB2:OP871961,PB1:OP871962,PA:OP871963,HA:OP871964,NP:OP871965,NA:OP871966,M:OP871967,NS:OP871968), A/New York City/PV00190/2017 (EPI_ISL_15842288, PB2:OP871945,PB1:OP871946,PA:OP871947,HA:OP871948,NP:OP871949,NA:OP871950,M:OP871951,NS:OP871952), A/New York City/PV02895/2019 (EPI_ISL_15842298, PB1:MW650793,HA:MW650794,PB2:MW650795,M:MW650796,NP:MW650797,PA:MW650798,NA:MW650799,NS:MW650800), A/New York City/PV01536/2018 (EPI_ISL_4072967, PB2:MW651804,NS:MW651805,PB1:MW651806,PA:MW651807,NP:MW651808,HA:MW651809,M:MW651810,NA:MW651811), A/New York City/PV00072/2017 (EPI_ISL_15842308, PB2:OP872041,PB1:OP872042,PA:OP872043,HA:OP872044,NP:OP872045,NA:OP872046,M:OP872047,NS:OP872048), A/New York City/PV00009/2017 (EPI_ISL_15842318, PB2:OP872001,PB1:OP872002,PA:OP872003,HA:OP872004,NP:OP872005,NA:OP872006,M:OP872007,NS:OP872008), A/New York City/PV00098/2017 (EPI_ISL_15842329, PB2:OP871929,PB1:OP871930,PA:OP871931,HA:OP871932,NP:OP871933,NA:OP871934,M:OP871935,NS:OP871936), A/New York City/PV00046/2017 (EPI_ISL_15842341, PB2:OP872049,PB1:OP872050,PA:OP872051,HA:OP872052,NP:OP872053,NA:OP872054,M:OP872055,NS:OP872056), A/New York City/PV01296/2018 (EPI_ISL_326915, PB2:OP913191,PB1:OP913192,PA:OP913193,HA:OP913194,NP:OP913195,NA:OP913196,M:OP913197), A/New York City/PV02516/2019 (EPI_ISL_15842351, NA:MW650801,NS:MW650802,PB1:MW650803,PB2:MW650804,HA:MW650805,PA:MW650806,M:MW650807,NP:MW650808), A/New York City/PV03878/2019 (EPI_ISL_15842352, PB2:OP871897,PB1:OP871898,PA:OP871899,HA:OP871900,NP:OP871901,NA:OP871902,M:OP871903,NS:OP871904), A/New York City/PV00099/2017 (EPI_ISL_4072918, PA:MW651788,PB2:MW651789,HA:MW651790,PB1:MW651791,NS:MW651792,NA:MW651793,NP:MW651794,M:MW651795), A/New York City/PV00105/2017 (EPI_ISL_15842353, PB2:OP871953,PB1:OP871954,PA:OP871955,HA:OP871956,NP:OP871957,NA:OP871958,M:OP871959,NS:OP871960), A/Kansas/14/2017 (EPI_ISL_403059), A/Singapore/INFIMH-16-0019/2016 (EPI_ISL_13046488), A/Switzerland/8060/2017 (EPI_ISL_332305), A/Switzerland/9715293/2013 (EPI_ISL_230377), A/Hong Kong/4801/2014 (EPI_ISL_389028) and A/Aichi/2/1968 (EPI_ISL_123225). Source data are provided with this paper.

## Source data

Source Data
