# Supplementary material for: Antibodies targeting the neuraminidase active site inhibit influenza H3N2 viruses with an S245N glycosylation site
Source: Nat Commun. 2022 Dec 21;13:7864. doi: 10.1038/s41467-022-35586-7 (PMC9772378; doi:10.1038/s41467-022-35586-7)
Supplement: Supplementary file 1 — Supplementary Information [file 41467_2022_35586_MOESM1_ESM.pdf]

Antibodies targeting the neuraminidase active site inhibit influenza H3N2 viruses with an S245N glycosylation site

Supplementary information

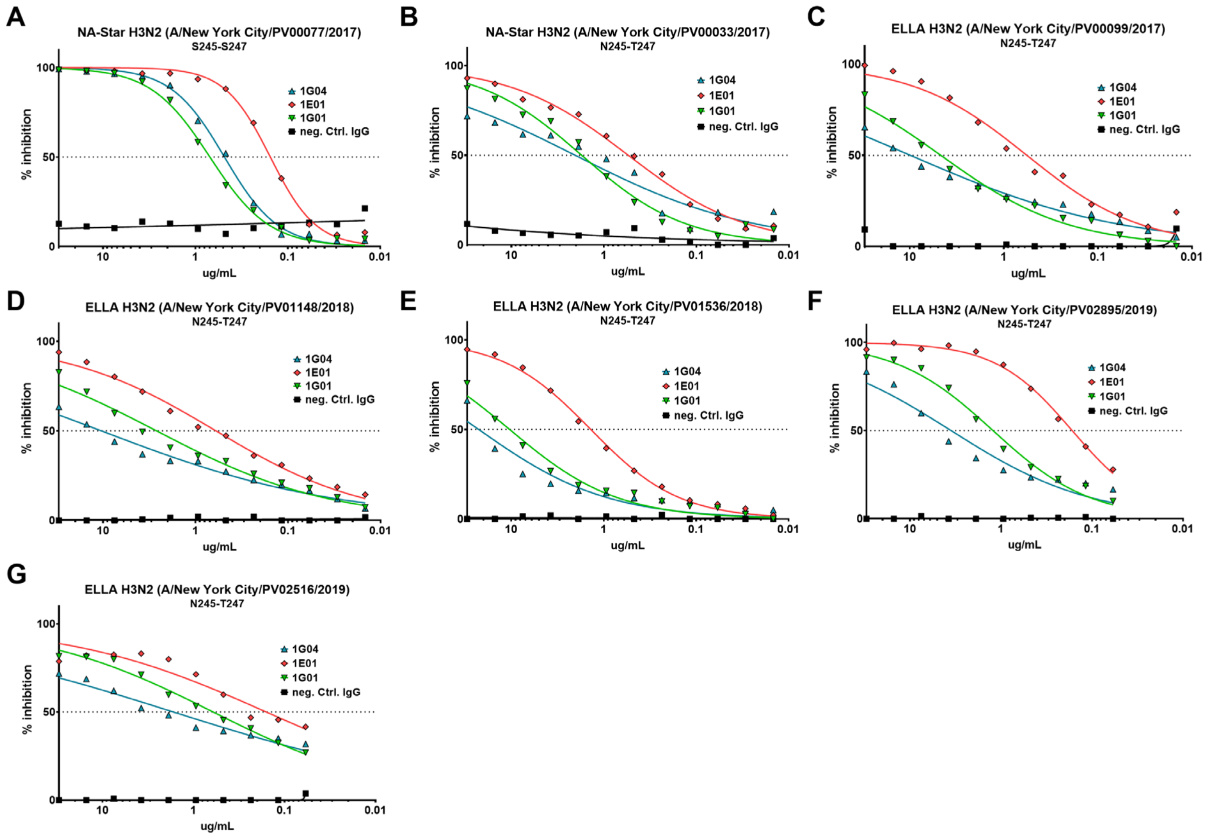

Supplementary Fig. 1. Neuraminidase inhibition of primary H3N2 virus isolates.
Inhibition curves as measured in NA-Star assay (**A-B**) and ELLAs (**C-G**) for recent, clinical H3N2 viruses as indicated. The antibodies were tested against the viruses in duplicate and the average of the two measurements was used to for graphing. Source data are provided as a Source Data file.

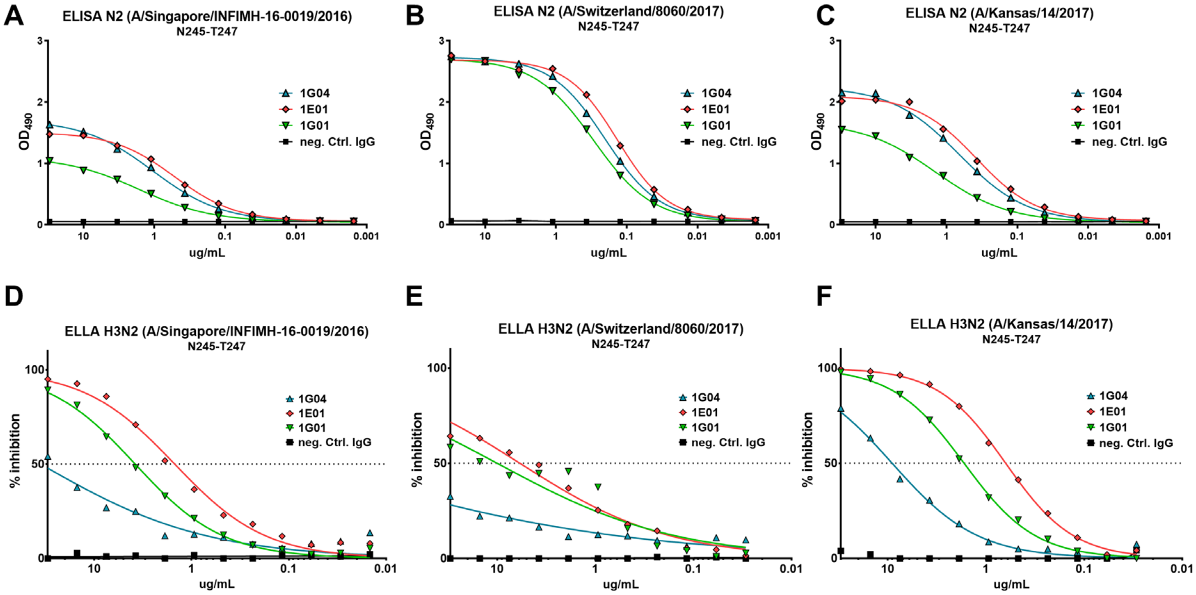

Supplementary Fig. 2. Antibody binding to recombinant neuraminidases of vaccine strain viruses and neuraminidase inhibition of the same viruses in ELLAs.

Binding curves as measured in ELISAs for N2 NA of A/Singapore/INFIMH-16-0019/2016 (**A**), A/Switzerland/8060/2017 (**B**) and A/Kansas/14/2017 (**C**). Neuraminidase inhibition curves for the same viruses are shown (**D-F**). The antibodies were tested against the viruses or recombinant proteins in duplicate and the average of the two measurements was used for graphing. Source data are provided as a Source Data file.

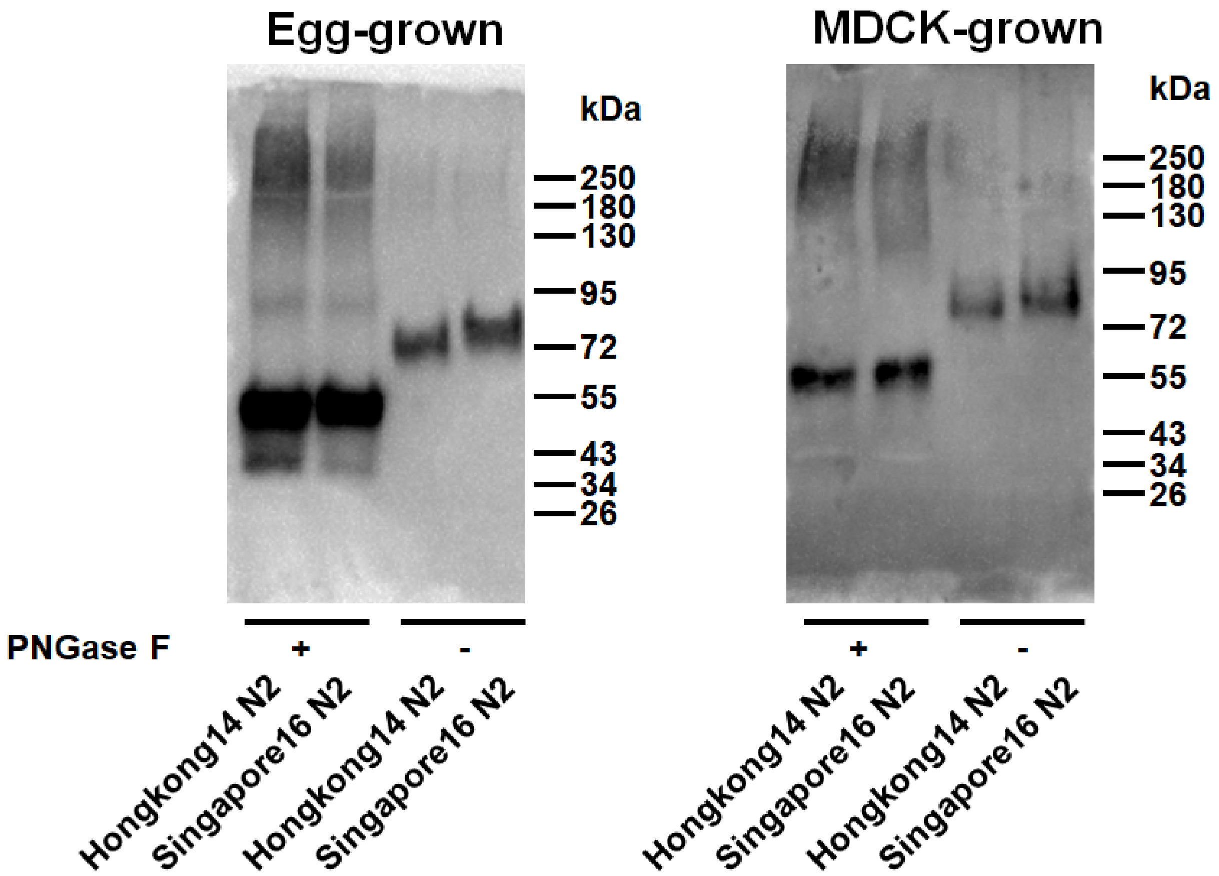

Supplementary Fig. 3. Glycosylation of influenza viruses.

The different systems (chicken embryonated eggs and MDCK cells) to grow viruses are indicated. Cell-grown and egg-grown viruses that lack the glycosylation site at NA Asn245 (HongKong14) were compared to cell-grown and egg-grown viruses that harbor the glycan (Singapore16) following PNGase treatment. PNGase treatment (+) or lack thereof (-) of virus preparations or viruses is indicated. Differences in glycan expression were assessed via Western blotting using anti-N2 guinea pig sera for detection. The experiment was performed once with these exact conditions. Comparable results were generated twice with slightly different conditions. Uncropped source data of the blot is provided on the final page of this document.

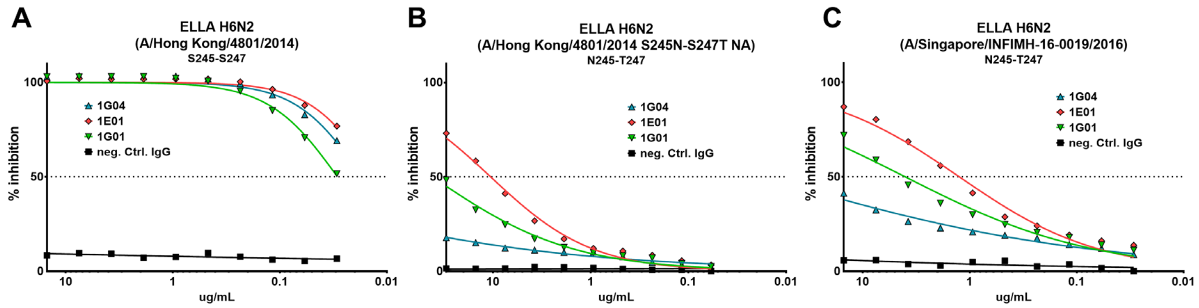

Supplementary Fig. 4. Neuraminidase inhibition of reassortant H6N2 viruses.

Inhibition curves as measured in ELLAs for reassortant H6N2 viruses that express the N2 NA of wild-type A/Hong Kong/4801/2014 (**A**), A/Hong Kong/4801/2014 with amino acid changes at position 245/247 (**B**) and A/Singapore/INFIMH-16-0019/2016 (**C**). The antibodies were tested against the viruses in duplicate and the average of the two measurements was used for graphing. Source data are provided as a Source Data file.

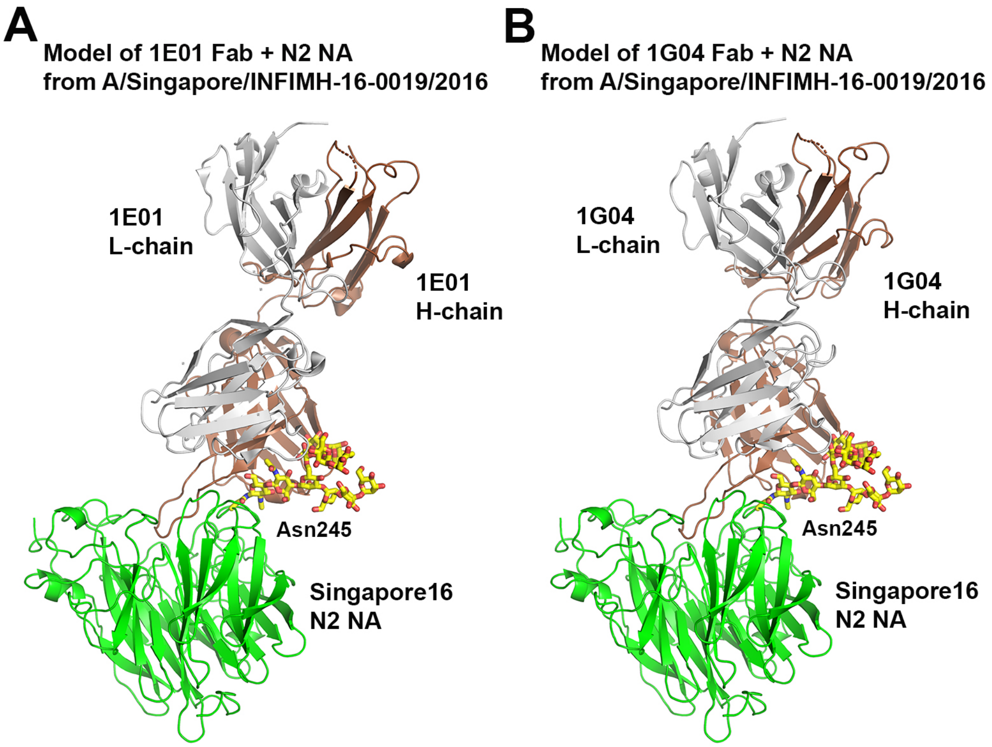

Supplementary Fig. 5. Model of the complex of Singapore16 N2 NA with 1E01 Fab (A) and 1G04 Fab (B). The Singapore16 N2 NA was modeled with a mannose-9 glycan at Asn245. The 1E01 and 1G04 Fabs are able to interact with the N2 NA. The NA protomer is colored in green with Asn245 and attached glycans are shown in sticks and colored with yellow carbon, red oxygen, and blue nitrogen atoms. The 1E01 and 1G04 Fabs are colored in brown (heavy chain) and grey (light chain).

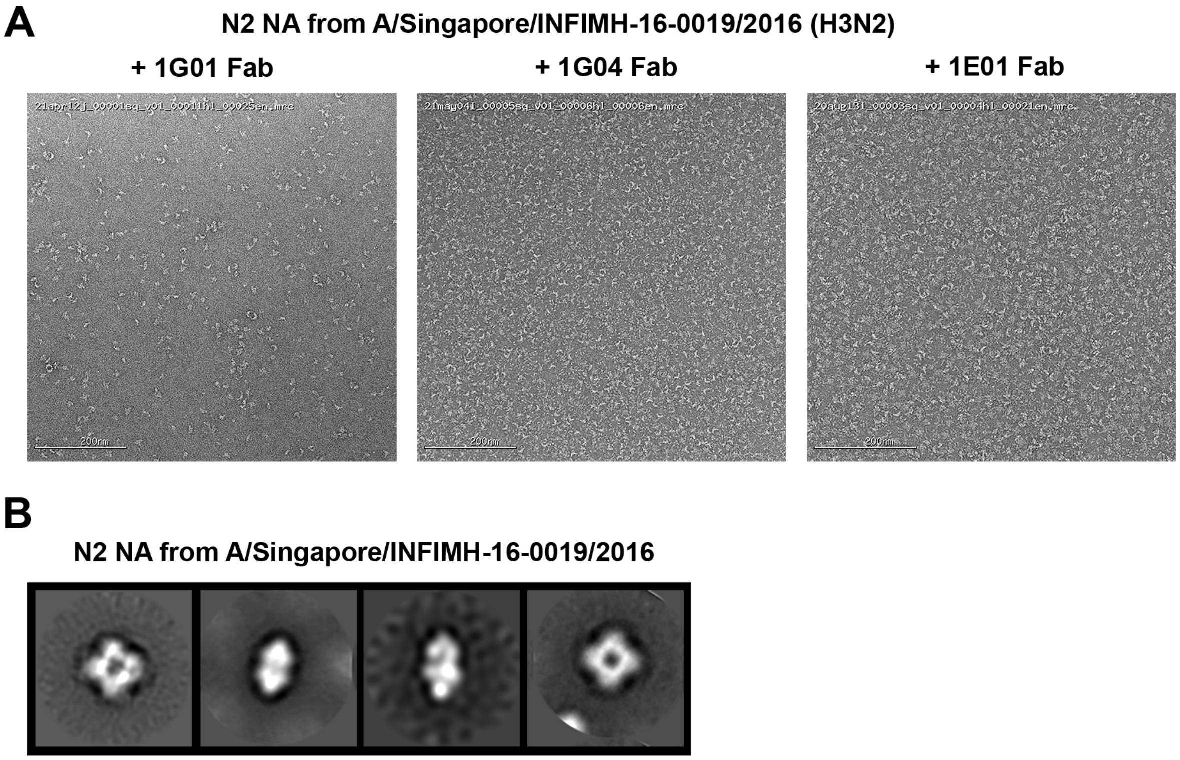

Supplementary Fig. 6. Negative stain electron microscopy of Singapore16 N2 NA and complexes with 1G01, 1G04 and 1E01 Fabs.

(A) Negative stain electron microscopy images for Singapore16 N2 NA in complex with 1G01 Fab (204 micrographs, 77,452 particles picked, 4,602 particles selected into 3D map, box size 144 at 1.98 Å/pix, final map resolution 16.0 Å using C4 symmetry), 1G04 Fab (393 micrographs, 169,348 particles picked, 22,681 particles selected into 3D map, box size 160 at 1.98 Å/pix, final map resolution 13.2 Å using C4 symmetry) and 1E01 Fab (310 micrographs, 180,086 particles picked, 131,777 particles selected into 3D map, box size 160 at 1.77 Å/pix, final map resolution 15.0 Å using C1 symmetry). The experiment was performed once. (B) Two-dimensional class averages of unliganded Singapore16 N2 NA.

```
                                                    118
Japan57 N2          VEYRNWSKPQCQITGFAPFSKDNSIRLSAGGDIWVTREPYVSCDPGKCYQFALGQGTTL
HongKong14 N2       AEYRNWSKPQCGITGFAPFSKDNSIRLSAGGDIWVTREPYVSCDPDKCYQFALGQGTTL
Singapore16 N2      AEYRNWSKPQCGITGFAPFSKDNSIRLSAGGDIWVTREPYVSCDPDKCYQFALGQGTTL
                    .********** *********************************.*************
                            151                                       198
Japan57 N2         DNKHSNGTIHDRIPHRTLLMNELGVPFHLGTKQVCVAWSSSSCHDGKAWLHVCVTGDDRN
HongKong14 N2      NNVHSNNKVRDRTPYRTLLMNELGVPFHLGTKQVCIAWSSSSCHDGKAWLHVCITGDDKN
Singapore16 N2     NNVHSNNTVRDRTPYRTLLMNELGVPFHLGTKQVCIAWSSSSCHDGKAWLHVCITGDDKN
                   :* ***..::** *:*******************:*****************:****:*
                                 220 224227                 245 249       259
Japan57 N2         ATASFIYDGRLVDSIGSWSQNILRTQESECVCINGTCTVVMTDGSASGRADTRILFIKEG
HongKong14 N2      ATASFIYNGRLVDSVVSWSKDILRTQESECICINGTCTVVMTDGSASGKADTKILFIEEG
Singapore16 N2     ATASFIYNGRLIDSVVSWSKDILRTQESECVCINGTCTVVMTDGNATGKADTKILFIEEG
                   *******:***:**: ***::*********:*************.*:*:***:****:**
                                                 296
Japan57 N2         KIVHISPLSGSAQHIEECSCYPRYPDVRCICRDNWKGSNRPVIDINMEDYSIDSSYVCSG
HongKong14 N2      KIVHTSTLSGSAQHVEECSCYPRYPGVRCVCRDNWKGSNRPIVDINIKDHSIVSSYVCSG
Singapore16 N2     KIVHTSKLSGSAQHVEECSCYPRYPGVRCVCRDNWKGSNRPIVDINIKDHSIVSSYVCSG
                   **** * *******:**********.***:***********::***::*:** *******

Japan57 N2         LVGDTPRNDDSSSNSNCRDPNNERGNPGVKGWAFDNGDDVWMGRTISKDSRSGYETFKVI
HongKong14 N2      LVGDTPRKNDSSSSSHCLDPNNEEGGHGVKGWAFDDGNDVWMGRTINETSRLGYETFKVI
Singapore16 N2     LVGDTPRKNDSSSSSHCLNPNNEEGGHGVKGWAFDDGNDVWMGRTINETSRLGYETFKVV
                   *******::****.*:* :****.*. ********:*:********.: ** *******:
                                          406                       432
Japan57 N2         GGWSTPNSKSQVNRQVIVDNNNWSGYSGIFSVEGKSCINRCFYVELIRGRPQETRVWWTS
HongKong14 N2      EGWSNPKSKLQTNRQVIVDRGDRSGYSGIFSVEGKSCINRCFYVELIRGRKEETEVLWTS
Singapore16 N2     EGWSNPKSKLQINRQVIVDRGDRSGYSGIFSVEGKSCINRCFYVELIRGRKEETEVLWTS
                    ***.*:** * *******..: *************************** :**.* ***

Japan57 N2         NSIVVFCGTSGTYGTGSWPDGANINFMPI
HongKong14 N2      NSIVVFCGTSGTYGTGSWPDGADLNLMPI
Singapore16 N2     NSIVVFCGTSGTYGTGSWPDGADLNLMHI
                   **********************::*:* *
```

Supplementary Fig. 7. Sequence alignment of N2 NAs with the 1E01 epitope footprint residues in Japan57 N2 NA highlighted in cyan (from PDB 6Q20). Epitope residues that differ in HongKong14 N2 and Singapore16 N2 NAs are highlighted in red, although some of these are conservative substitutions (e.g. R to K, N to D, Q to E).

```
                                                           118             134
Cali04 N1                SVKLAGNSSLCPVSGWAIYSKDNSVRIGSKGDVFVIREPFISCSPLECRTFFLTQGALL
HongKong14 N2            AEYRNWSKPQCGITGFAPFSKDNSIRLSAGGDIWVTREPYVSCDPDKCYQFALGQGTTL
Singapore16 N2           AEYRNWSKPQCGITGFAPFSKDNSIRLSAGGDIWVTREPYVSCDPDKCYQFALGQGTTL
                         :     ... * ::*:* :*****:*:.: **::* ***::**.* :*  * * **: *

                               150   156                    178                197
Cali04 N1               NDKHSNGTIKDRSPYRTLMSCPIGEVPSPYNSRFESVAWSASACHDGINWLTIGISGPDN
HongKong14 N2           NNVHSNNKVRDRTPYRTLLMNELG-VPFHLGTKQVCIAWSSSSCHDGKAWLHVCITGDDK
Singapore16 N2          NNVHSNNTVRDRTPYRTLLMNELG-VPFHLGTKQVCIAWSSSSCHDGKAWLHVCITGDDK
                        *: ***..::**:*****:   :* **   .::  .:***:*:****  ** : *:* *:

                                          220 224                245
Cali04 N1               GAVAVLKYNGIITDTIKSWRNNILRTQESECACVNGSCFTVMTDGPSNGQASYKIFRIEK
HongKong14 N2           NATASFIYNGRLVDSVVSWSKDILRTQESECICINGTCTVVMTDGSASGKADTKILFIEE
Singapore16 N2          NATASFIYNGRLIDSVVSWSKDILRTQESECVCINGTCTVVMTDGNATGKADTKILFIEE
                        .*.* : *** : *:: ** ::********* *:**:* .***** :.*:*. **: **:

                                      276
Cali04 N1               GKIVKSVEMNAPNYHYEECSCYPDSSEITCVCRDNWHGSNRPWVSFNQNLEYQIG-YICS
HongKong14 N2           GKIVHTSTLSGSAQHVEECSCYPRYPGVRCVCRDNWKGSNRPIVDINIKDHSIVSSYVCS
Singapore16 N2          GKIVHTSKLSGSAQHVEECSCYPRYPGVRCVCRDNWKGSNRPIVDINIKDHSIVSSYVCS
                        ****::  :...  * *******  . : *******:***** *.:* : .  :. *:**

                                                 347                     371
Cali04 N1               GIFGDNPRPNDKTGS---CGPVSSNGANGVKGFSFKYGNGVWIGRTKSISSRNGFEMIWD
HongKong14 N2           GLVGDTPRKNDSSSSSHCLDPNNEEGGHGVKGWAFDDGNDVWMGRTINETSRLGYETFKV
Singapore16 N2          GLVGDTPRKNDSSSSSHCLNPNNEEGGHGVKGWAFDDGNDVWMGRTINETSRLGYETFKV
                        *:.**.** **.:.*    .* ..:*.:****::*. **.**:*** . :** *:* :

                                                406                          431
Cali04 N1               PNGWTGTDNNFSIKQDIVG-INEWSGYSGSFVQHPELTGLDCIRPCFWVELIRGRPKE-N
HongKong14 N2           IEGWSNPKSKLQTNRQVIVDRGDRSGYSGIFSVE----GKSCINRCFYVELIRGRKEETE
Singapore16 N2          VEGWSNPKSKLQINRQVIVDRGDRSGYSGIFSVE----GKSCINRCFYVELIRGRKEETE
                         :**:....::. :::::   .: ***** *  .    * .**. **:******* :* :

                                          456
Cali04 N1               TIWTSGSSISFCGVNSDTVGWSWPDGAELPFTI
HongKong14 N2           VLWTSNSIVVFCGTSGTYGTGSWPDGADLNLMP
Singapore16 N2          VLWTSNSIVVFCGTSGTYGTGSWPDGADLNLMH
                        .:***.* : ***...     ******:* :
```

Supplementary Fig. 8. Sequence alignment of N2 NAs with the 1G01 epitope footprint residues in Cali04 N1 NA highlighted in cyan (from PDB 6Q23). Epitope residues that differ in HongKong14 N2 and Singapore16 N2 NAs are highlighted in red.

```
                                                       118
Hunan16 N9           RNFNNLTKGLCTINSWHIYGKDNAVRIGESSDVLVTREPYVSCDPDECRFYALSQGTTI
HongKong14 N2        AEYRNWSKPQCGITGFAPFSKDNSIRLSAGGDIWVTREPYVSCDPDKCYQFALGQGTTL
Singapore16 N2       AEYRNWSKPQCGITGFAPFSKDNSIRLSAGGDIWVTREPYVSCDPDKCYQFALGQGTTL
                       ::.* :*  * *..:  :.***::*:. ..*: ************:*  :**.****:

                           149                                               198
Hunan16 N9           RGKHSNGTIHDRSQYRALISWPLSSPPTVHNSRVECIGWSSTSCHDGKSRMSICISGPNN
HongKong14 N2        NNVHSNNKVRDRTPYRTLLMNELGVPFHLG-TKQVCIAWSSSSCHDGKAWLHVCITGDDK
Singapore16 N2       NNVHSNNTVRDRTPYRTLLMNELGVPFHLG-TKQVCIAWSSSSCHDGKAWLHVCITGDDK
                     .. ***..::**: **:*:   *. *  :  ::  **.***:******: : :**:* ::

                                       220 224                   245
Hunan16 N9           NASAVVWYNRRPVAEINTWARNILRTQESECVCHNGVCPVVFTDGPATGPADTRIYYFKE
HongKong14 N2        NATASFIYNGRLVDSVVSWSKDILRTQESECICINGTCTVVMTDGSASGKADTKILFIEE
Singapore16 N2       NATASFIYNGRLIDSVVSWSKDILRTQESECVCINGTCTVVMTDGNATGKADTKILFIEE
                     **:* . ** * : .: :*:::*********:* **.*.**:*** *:* ***:* :::*

                                   276                  295
Hunan16 N9           GKILKWESLTGTAKHIEECSCYGERTGITCTCRDNWQGSNRPVVQIDPVAMTHTSQYICS
HongKong14 N2        GKIVHTSTLSGSAQHVEECSCYPRYPGVRCVCRDNWKGSNRPIVDINIKDHSIVSSYVCS
Singapore16 N2       GKIVHTSKLSGSAQHVEECSCYPRYPGVRCVCRDNWKGSNRPIVDINIKDHSIVSSYVCS
                     ***:: ..*:*:*:*:****** . .*: *.*****:*****:*:*:    : .*.*:**

                                            345                      370
Hunan16 N9           PVLTDSPRPNDPNIG-KCNDPYPGNNNNGVKGFSYLDGANTWLGRTISTASRSGYEMLKV
HongKong14 N2        GLVGDTPRKNDSSSSSHCLDPNNEEGGHGVKGWAFDDGNDVWMGRTINETSRLGYETFKV
Singapore16 N2       GLVGDTPRKNDSSSSSHCLNPNNEEGGHGVKGWAFDDGNDVWMGRTINETSRLGYETFKV
                      :: *:** **.. . :* :*   :..:****::: **  :.*:****. :** *** :**

                                             406                         432
Hunan16 N9           PNALTDDRSKPIQG-QTIVLNADWSGYSGSFMDYWAEGDCYRACFYVELIRGRPKEDKVW
HongKong14 N2        IEGWSNPKSKLQTNRQVIVDRGDRSGYSGIFSVEGKS--CINRCFYVELIRGRKEETEVL
Singapore16 N2       VEGWSNPKSKLQINRQVIVDRGDRSGYSGIFSVEGKS--CINRCFYVELIRGRKEETEVL
                      :. :: :**   . *.** ..* ***** *     .  * . ********** :* :*

Hunan16 N9           WTSNSIVSMCSSTEFLGQWNWPDGAKIEYFL
HongKong14 N2        WTSNSIVVFCGTSGTYGTGSWPDGADLNLMP
Singapore16 N2       WTSNSIVVFCGTSGTYGTGSWPDGADLNLMH
                     ******* :*.::   *  .*****.:: :
```

Supplementary Fig. 9. Sequence alignment of N2 NAs with the 1G04 epitope footprint residues in Hunan16 N9 NA highlighted in cyan (from PDB 6Q1Z). Epitope residues that differ in HongKong14 N2 and Singapore16 N2 NAs are highlighted in red.

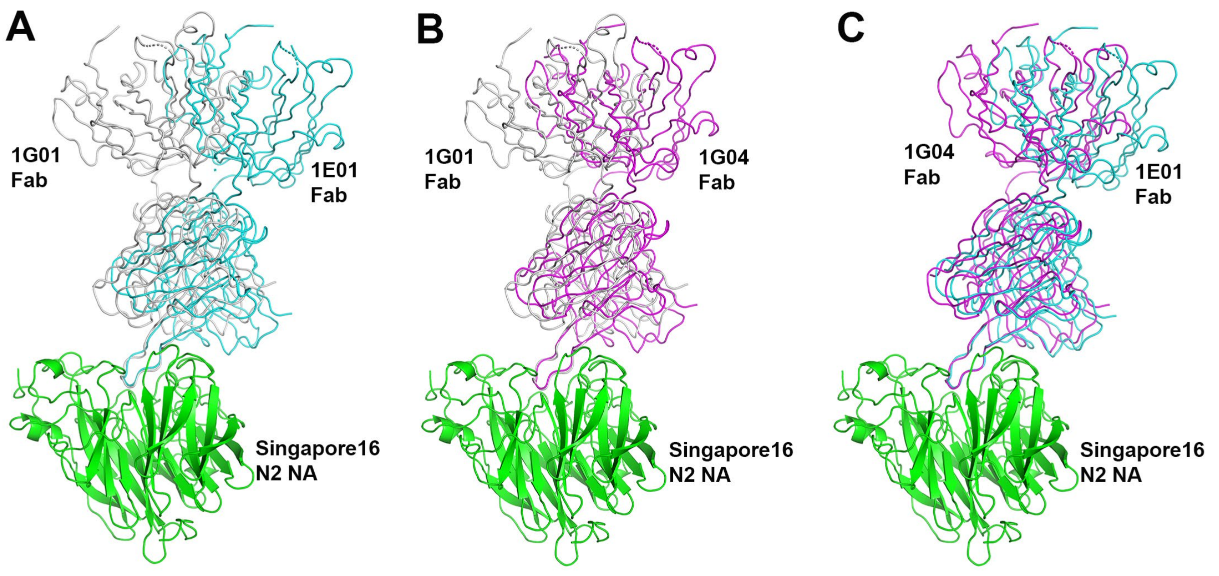

Supplementary Fig. 10. Superimpositions of models of the complexes of Singapore16 N2 NA with 1G01, 1E01 and 1G04 Fabs. (A) Superimposition of the N2 NA models from its complexes with 1G01 and 1E01 Fabs. (B) Superimposition of the N2 NA models from its complexes with 1G01 and 1G04 Fabs. (C) Superimposition of the N2 NA models from its complexes with 1E01 and 1G04 Fabs. 1G01, 1E01 and 1G04 appear to bind the N2 NA with slightly different angles of approach.

Source data

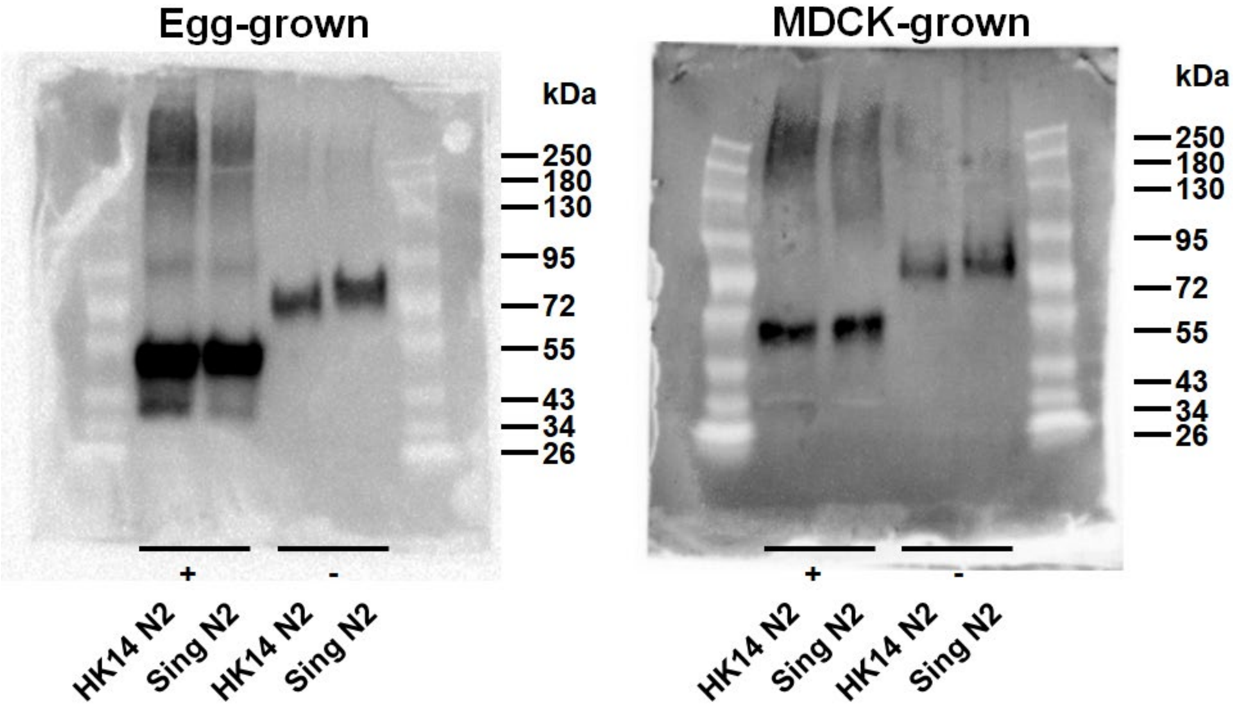

Uncropped version of Supplementary Figure 3.
