# Supplementary material for: Antibodies targeting the neuraminidase active site inhibit influenza H3N2 viruses with an S245N glycosylation site
Source: Nat Commun. 2022 Dec 21;13:7864. doi: 10.1038/s41467-022-35586-7 (PMC9772378; doi:10.1038/s41467-022-35586-7)
Supplement: Supplementary file 2 — Reporting Summary [file 41467_2022_35586_MOESM2_ESM.pdf]

nature portfolio

Corresponding author(s): Florian Krammer

Last updated by author(s): Nov 8, 2022

Reporting Summary

Nature Portfolio wishes to improve the reproducibility of the work that we publish. This form provides structure for consistency and transparency in reporting. For further information on Nature Portfolio policies, see our Editorial Policies and the Editorial Policy Checklist.

Statistics

For all statistical analyses, confirm that the following items are present in the figure legend, table legend, main text, or Methods section.

n/a	Confirmed	
☐	☒	The exact sample size (*n*) for each experimental group/condition, given as a discrete number and unit of measurement
☐	☒	A statement on whether measurements were taken from distinct samples or whether the same sample was measured repeatedly
☒	☐	The statistical test(s) used AND whether they are one- or two-sided<br>*Only common tests should be described solely by name; describe more complex techniques in the Methods section.*
☒	☐	A description of all covariates tested
☒	☐	A description of any assumptions or corrections, such as tests of normality and adjustment for multiple comparisons
☐	☒	A full description of the statistical parameters including central tendency (e.g. means) or other basic estimates (e.g. regression coefficient) AND variation (e.g. standard deviation) or associated estimates of uncertainty (e.g. confidence intervals)
☒	☐	For null hypothesis testing, the test statistic (e.g. *F*, *t*, *r*) with confidence intervals, effect sizes, degrees of freedom and *P* value noted<br>*Give P values as exact values whenever suitable.*
☒	☐	For Bayesian analysis, information on the choice of priors and Markov chain Monte Carlo settings
☒	☐	For hierarchical and complex designs, identification of the appropriate level for tests and full reporting of outcomes
☒	☐	Estimates of effect sizes (e.g. Cohen's *d*, Pearson's *r*), indicating how they were calculated

Our web collection on statistics for biologists contains articles on many of the points above.

Software and code

Policy information about availability of computer code

Data collection	LabScan software (GE Healthcare) was used for Western blots, Leginon Software (NRAMM) was used for image acquisition for EM, Gen 3.11 software (BioTek) was used for plate-based assays.
Data analysis	Structural models were generated in MODELLER and Coot. Binding/functional data were analyzed in Microsoft Excel and GraphPad Prism 7 and 8. Blots were analyzed using LabScan software. All programs are commercially available or open source.

For manuscripts utilizing custom algorithms or software that are central to the research but not yet described in published literature, software must be made available to editors and reviewers. We strongly encourage code deposition in a community repository (e.g. GitHub). See the Nature Portfolio guidelines for submitting code & software for further information.

Data

Policy information about availability of data

All manuscripts must include a data availability statement. This statement should provide the following information, where applicable:

- Accession codes, unique identifiers, or web links for publicly available datasets
- A description of any restrictions on data availability
- For clinical datasets or third party data, please ensure that the statement adheres to our policy

Data supporting our work are available in the paper and Supplementary Figures 1-10. Source data are provided with this paper.

Human research participants

Policy information about studies involving human research participants and Sex and Gender in Research.

Reporting on sex and gender	N/A
Population characteristics	N/A
Recruitment	N/A
Ethics oversight	N/A

Note that full information on the approval of the study protocol must also be provided in the manuscript.

Field-specific reporting

Please select the one below that is the best fit for your research. If you are not sure, read the appropriate sections before making your selection.

☒ Life sciences ☐ Behavioural & social sciences ☐ Ecological, evolutionary & environmental sciences

For a reference copy of the document with all sections, see nature.com/documents/nr-reporting-summary-flat.pdf

Life sciences study design

All studies must disclose on these points even when the disclosure is negative.

Sample size	The group size was n=5 for mouse experiments except for the control group in the middle panel where the n was 4 for the control group. The sample size for mice was chosen on previous experience since it typically allows to see differences in weight loss and survival. For testing monoclonal antibodies the sample size was one due to the very nature of monoclonal antibodies.
Data exclusions	A single data point in Suppl. Figure 2E was excluded from one of the duplicates in one of the mAbs. This was done because the point was identified as an outlier upon visual inspection of the curve. This sometimes happens for single spots in 96-well plates due to technical errors of the automated washer.
Replication	Binding and functional assays were performed once in duplicates and the average of the technical duplicates was graphed. The singlets did yield comparable results. The Western blotting experiment was performed once using these exact conditions but comparable results were generated several times with slightly different conditions.
Randomization	Samples were not randomized since only a small number of mAbs were characterized and randomization would have been impractical and would not have added value since these were simple comparisons between three mAbs and a negative control. A set of experiments including all three mAbs and the controls were done together, minimizing any impact of covariates. The only non-mAb experiment involves the Western blot in Supplementary Figure 3. Similar to the mAb experiments, randomization would have been impractical and irrelevant here for exactly the same reasons.
Blinding	Operators were not blinded since only a small number of mAbs were characterized and blinding would have been impractical and would not have added value. This also applies to the Western blot in Supplementary Figure 3.

Reporting for specific materials, systems and methods

We require information from authors about some types of materials, experimental systems and methods used in many studies. Here, indicate whether each material, system or method listed is relevant to your study. If you are not sure if a list item applies to your research, read the appropriate section before selecting a response.

Materials & experimental systems

n/a	Involved in the study	
☐	☒	Antibodies
☐	☒	Eukaryotic cell lines
☒	☐	Palaeontology and archaeology
☐	☒	Animals and other organisms
☒	☐	Clinical data
☒	☐	Dual use research of concern

Methods

n/a	Involved in the study	
☒	☐	ChIP-seq
☒	☐	Flow cytometry
☒	☐	MRI-based neuroimaging

Antibodies

Antibodies used	primary antibodies: mAbs 1G01, 1G04 and 1E01. Since these antibodies are our antibodies that we characterize here, they do not have any catalogue number. Furthermore, concentrations/dilutions used are specified whenever they are used. This is obviously indicated in the figures.<br>Secondary antibodies: anti-human IgG (Fab specific) horseradish peroxidase antibody (HRP; Sigma, #A0293), donkey anti-guinea pig IgG-horseradish peroxidase (IgG-HRP; EMD, AP193P)
Validation	mAbs 1G01, 1G04 and 1E01 were characterized and validated in Stadlbauer et al., Science, 2019<br>Secondary antibodies were validated by the manufacturer.

Eukaryotic cell lines

Policy information about cell lines and Sex and Gender in Research

Cell line source(s)	Madin Darby canine kidney (MDCK) and 293T cell lines were acquired from ATCC. 293F cells were acquired from Thermo Fisher. Sf9 and BTI-TN-5B1- 4 (High Five) were sourced from the Vienna Institute of Biotechnology/University of Natural Resources and Life Sciences.
Authentication	None of the cell lines were authenticated.
Mycoplasma contamination	Cell lines were not tested for mycoplasma contamination.
Commonly misidentified lines<br>(See ICLAC register)	No commonly misidentifed cell lines were used.

Animals and other research organisms

Policy information about studies involving animals; ARRIVE guidelines recommended for reporting animal research, and Sex and Gender in Research

Laboratory animals	6-8 week old female DBA/2J mice were purchased from The Jackson Laboratory.
Wild animals	No wild animals were used.
Reporting on sex	Only female mice were used for the antibody evaluation. For mAb testing, no sex differences are expected in mice.
Field-collected samples	No field-collected samples were used.
Ethics oversight	Animal experiments were conducted in compliance with protocols approved by the Icahn School of Medicine at Mount Sinai's Institutional Animal Care and Use Committee.

Note that full information on the approval of the study protocol must also be provided in the manuscript.
